# Combined target site (*kdr*) mutations play a primary role in highly pyrethroid resistant phenotypes of *Aedes aegypti* from Saudi Arabia

**DOI:** 10.1186/s13071-017-2096-6

**Published:** 2017-03-27

**Authors:** Ashwaq M. Al Nazawi, Jabir Aqili, Mohammed Alzahrani, Philip J. McCall, David Weetman

**Affiliations:** 1 Department of Vector Biology, Liverpool School of Tropical Medicine, Pembroke Place, Liverpool, L3 5QA UK; 2 Saudi Ministry of Health, Riyadh, Saudi Arabia

**Keywords:** Dengue, Saudi Arabia, *Aedes aegypti*, Insecticide resistance, Piperonyl butoxide (PBO), Knockdown resistance

## Abstract

**Background:**

Pyrethroid resistance is a threat to effective vector control of *Aedes aegypti*, the vector of dengue, Zika and other arboviruses, but there are many major knowledge gaps on the mechanisms of resistance. In Jeddah and Makkah, the principal dengue-endemic areas of Saudi Arabia, pyrethroids are used widely for *Ae. aegypti* control but information about resistance remains sparse, and the underlying genetic basis is unknown. Findings from an ongoing study in this internationally significant area are reported here.

**Methods:**

*Aedes aegypti* collected from each city were raised to adults and assayed for resistance to permethrin, deltamethrin (with and without the synergist piperonyl butoxide, PBO), fenitrothion, and bendiocarb. Two fragments of the voltage-gated sodium channel (*Vgsc*), encompassing four previously identified mutation sites, were sequenced and subsequently genotyped to determine associations with resistance. Expression of five candidate genes (*CYP9J10*, *CYP9J28*, *CYP9J32*, *CYP9M6*, *ABCB4*) previously associated with pyrethroid resistance was compared between assay survivors and controls.

**Results:**

Jeddah and Makkah populations exhibited resistance to multiple insecticides and a similarly high prevalence of resistance to deltamethrin compared to a resistant Cayman strain, with a significant influence of age and exposure duration on survival*.* PBO pre-exposure increased pyrethroid mortality significantly in the Jeddah, but not the Makkah strain. Three potentially interacting *Vgsc* mutations were detected: V1016G and S989P were in perfect linkage disequilibrium in each strain and strongly predicted survival, especially in the Makkah strain, but were in negative linkage disequilibrium with 1534C, though some females with the *Vgsc* triple mutation were detected. The candidate gene *CYP9J28* was significantly over-expressed in Jeddah compared to two susceptible reference strains, but none of the candidate genes was consistently up-regulated to a significant level in the Makkah strain.

**Conclusions:**

Despite their proximity, Makkah and Jeddah exhibit significant differences in pyrethroid resistance phenotypes, with some evidence to suggest a different balance of mechanisms, for example with more impact associated with CYP450s in the Jeddah strain, and the dual *kdr* mutations 989P and 1016G in the more resistant Makkah strain. The results overall demonstrate a major role for paired target site mutations in pyrethroid resistance and highlight their utility for diagnostic monitoring.

**Electronic supplementary material:**

The online version of this article (doi:10.1186/s13071-017-2096-6) contains supplementary material, which is available to authorized users.

## Background

**Fig. 1:**
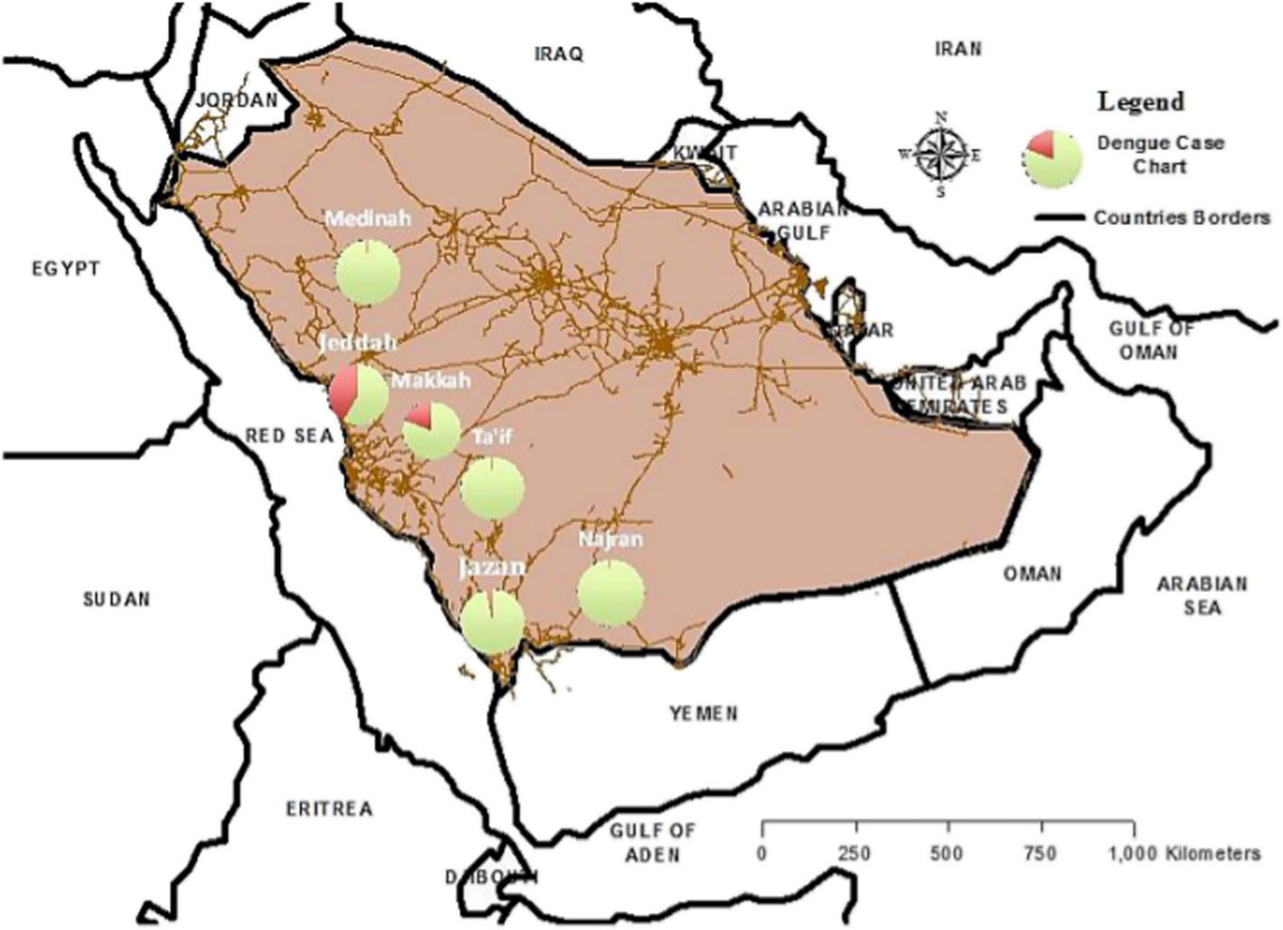
Dengue fever cases in cities of Saudi Arabia from 2013–2015

Dengue virus is transmitted by the bite of *Aedes* mosquitoes, especially *Ae. aegypti,* has recently increased dramatically in prevalence and now affects more than 100 tropical and sub-tropical countries, with half of the world’s population at risk [1, 2]. Although a licensed vaccine against the four dengue serotypes recently became available [3], and already is approved in some endemic countries [4, 5], protection is incomplete and unequal across serotypes [6]. Hence, *Aedes* control using insecticides will remain a key intervention for dengue prevention, especially given the added benefit of simultaneously targeting other *Aedes*-transmitted arboviruses including chikungunya and Zika. In Saudi Arabia, several areas are endemic regions for the dengue virus of which Jeddah and Makkah are by far the most important (Fig. 1). First reported in Jeddah in 1994, three serotypes DENG-1, DENG-2, DENG-3 [7] have been confirmed, and 9,096 cases were diagnosed between 2013 and 2015, with a further 3,035 in Makkah. Multiple insecticides are applied to target immature vector stages in Makkah and Jeddah, but pyrethroids are the most commonly used to control adult *Ae. aegypti* by indoor and outdoor space spraying. The efficacy of pyrethroid-based adult control has been found to be impacted by insecticide resistance in *Ae. aegypti* from diverse locations [8, 9]. Information on the insecticide resistance status of Saudi Arabian populations of *Ae. aegypti* remains limited [10, 11], and is absent for Jeddah, the primary national centre for dengue.

The most common mechanisms of insecticide resistance in *Ae. aegypti* involve target site alterations and metabolic resistance, comprising altered activity of enzymes from three superfamilies, esterases (CCEs), glutathione-s-transferases (GSTs) and cytochrome P450s (CYP450s), although several other enzyme families are likely to be involved [12]. Overexpression of *Ae. aegypti* CYP450s, especially from the CYP9 and CYP6 subfamilies has frequently been implicated in pyrethroid resistant phenotypes [13], and several overexpressed P450s have been shown to metabolise pyrethroids in vitro [14–16]. Pyrethroid and DDT target site resistance results from amino acid substitutions in the voltage-gated sodium channel (*Vgsc*) that usually reduce insecticide binding, although other mechanisms unrelated to binding are known [17, 18]. Some mutations in the insect *Vgsc* can prevent the normal action of pyrethroids and DDT (repetitive nerve firing, paralysis and death) leading to knockdown resistance (*kdr*) [18]. Multiple *kdr* mutations have been recorded in *Ae. aegypti* worldwide including G923V, L982W, I1011M/V, S989P, V1016G/I, F1534C and D1763Y [19, 20]. Of these, I1011M, V1016G/I [21], S989P [22] and F1534C [23, 24] either individually or in combination have been associated directly with pyrethroid resistance. The vast majority of studies of resistance mechanisms in *Ae. aegypti* are from The Americas and Asia [19, 25, 26], though a recent first study from West Africa identified the presence of target site resistance shared with American populations [27]. Knowledge of resistance mechanisms in the Middle East and their relationship with other regions is currently lacking. This paper investigates the resistance status of *Ae. aegypti* from Jeddah and Makkah, and focuses in particular on pyrethroid resistance and its underlying mechanisms as compared with established resistant and susceptible strains.

## Methods

### Mosquito strains, collection and rearing

The first collection in Jeddah (21°63'1.24"N, 39°19' 8.71"W) and Makkah (21°45'8.36"N, 39°78'6.98"E) was made in December to April (Makkah) and June to August 2015 (Jeddah). In both areas, 50 ovitraps were set targeting sites in which dengue cases have been reported. In Makkah, collections were made from private farms, brick factories, car and tyre shops, fuel stations and residential houses. Similarly, in Jeddah traps were set in buildings under construction, in shops and houses. All traps were checked every two days and collected after five days. The eggs were dried, preserved at room temperature in a sealed plastic bag and shipped to the Liverpool School of Tropical Medicine (LSTM). Egg batches were hatched, and larvae reared to adults on a diet of chinchilla pellets under insectary conditions of 27 °C and 70% relative humidity with a constant photoperiod (12 light:12 h dark). Adult females, 3–5 days old, from the first laboratory generation (i.e. the offspring of the adults from the egg collections, hereafter referred to as ‘F1 females’), which had been fed *ad libitum* on 10% sugar solution were used for phenotypic bioassays and characterization of molecular resistance mechanisms. The Cayman strain [23] used as a reference resistant strain and the standard strains New Orleans and Rockefeller as susceptible references (used for qPCR only); all strains were raised under the same conditions. A second, broader field collection in March-April 2016 targeted larvae present in water coolers, barrels, buckets and water containers such as under air conditioners and buildings under construction from a wide area within Jeddah and Makkah (Additional file 1: Figure S1). The larvae were reared at the local Municipal Insectaries on a diet of yeast under insectary conditions of 27 °C and 75% relative humidity with a constant photoperiod (12 light: 12 h dark) and females (hereafter referred to as ‘field females’), fed *ad libitum* on 10% sugar solution, were bioassayed at 3–5 days.

### Phenotypic assays

WHO tube bioassays were performed with a minimum of four replicates plus control, with approximately 25 females per tube. Using the standard WHO protocol [28], female mosquitoes were exposed to permethrin (0.75%), deltamethrin (0.05%), fenitrothion (1.0%) or bendiocarb (0.1%) for 60 min, under the insectary conditions described above, and then transferred to recovery tubes with access to 10% sugar solution. The mortality rate was recorded following 24 h exposure. Owing to logistical limitations, bendiocarb was not tested in the field females. F1 female mosquitoes were also exposed to deltamethrin after exposure to the synergist 4% piperonyl butoxide (PBO), which amongst other potential effects, is intended to block the action of CYP450s. We used the following procedure: 1 h control paper + 1 h deltamethrin, 1 h PBO + 1 h deltamethrin, 1 h PBO+ 1 h control; 2 h control paper, with mortality recorded after 24 h as before. The level of resistance to deltamethrin was investigated using bioassays with longer durations of exposure, in which the impact of age on mortality was also examined. Three-to-five and ten-day-old F1 adult females from the Jeddah, Makkah and Cayman strains were exposed for 1 h, 6 h or 8 h, with mortality recorded 24 h after the bioassay.

### Target site mutations

Genomic DNA was extracted from F0 females from the Jeddah (*N =* 26), and Makkah (*N =* 15) strains using Nexttec kits (Nexttec™, Biotechnologie GmbH, Hilgertshausen, Germany) according to the manufacturer’s instructions. PCR primers (IIS5-6 (3) F: 5′-ATC GCT TCC CGG ACA AAG AC-3′; IIS5-6 (3) R: 5′-GTT GGC GAT GTT CGA CTT GA-3′) were designed using Primer 3 [29, 30] to amplify *kdr* mutations in the sixth transmembrane segment of domain II of the *Vgsc*, which includes the resistance-associated codons 989, 1,011, 1,016. For the Makkah samples a segment of domain III that includes codon 1,534 we amplified exon 30 using the primers AaNa31F and AaNa31R [23]. PCR reactions were carried out in a 25 μl reaction volume containing 12.5 μl TaqRed Mix (Bioline, London, UK), 0.5 μl forward and reverse primers (10 μM) and 2 μl of DNA extract. Cycling conditions for the domain II primers were 95 °C for 5 min, 35 cycles of (94 °C for 30 s, 65 °C for 30 s, 72 °C for 1 min) and a 10 min final elongation step at 72 °C. For the domain III primers, conditions were the same except that the annealing temperature was 62 °C for 30 s. PCR products were purified using the QIAquick® PCR Purification Kit (Qiagen, Manchester, UK) and sequenced commercially (Source Bioscience, Rochdale, UK). Sequence data were assembled and aligned using Codon Code Aligner version 4.2.7. A TaqMan SNP genotyping assay for F1534C was designed and compared to an existing PCR tetraplex assay [23] and validated by comparison with DNA sequencing of field-caught Jeddah and Makkah mosquitoes. TaqMan reactions were performed in a 10 μl final volume containing 5 μl of TaqMan® Gene Expression SensiMix (Applied Biosystem, Foster city, USA), 0.125 μl primer/probe, 3.875 μl sterile water (Sigma, Gillingham, Dorset, UK) and 1 μl of DNA. Assays were run on an Agilent MX3000P qPCR thermal cycler under the following conditions: a 10 min cycle at 95 °C, and 40 cycles of 92 °C for 15 min and 60 °C for 1 min. Based on 21 samples, the concordance between the TaqMan SNP screening results and DNA sequencing analysis was 100%. Results were concordant between assays in 19 of 21 samples with remaining two genotypes giving an unclear score using the tetraplex method. To investigate the association between target site mutations and survival, F2 females from the Jeddah and Makkah strains were exposed to deltamethrin in WHO bioassays for either a standard 1 h duration and for longer periods of 4–6 h. Genotypes of females killed by 1 h of deltamethrin exposure (susceptible), were compared with survivors of the longer exposures.

### Gene expression

Expression profiles of genes previously associated with *Ae. aegypti* pyrethroid resistance, four CYP450 genes (*CYP9J10*, *CYP9J28*, *CYP9J32*, *CYP9M6*) and an ABC transporter (*ABCB4*) [16, 31, 32] (Additional file 2: Table S1) were assessed using quantitative reverse transcription PCR (qRT-PCR), in relation to two susceptible strains, New Orleans and Rockefeller. Total RNA was extracted from three pools of 5 female mosquitoes in each strain, which had not been exposed to insecticide, using the Ambion RNAqueous® Kit (Life Technologies, Paisley, UK), with the quantity of RNA yields assessed using a Nanodrop ND-1000 (Thermo Scientific, Delaware, USA). Synthesis of cDNA used Superscript III (Invitrogen, Carlsbad, CA, USA) according to the manufacturer’s guidelines, and cDNA was purified using a QIAquick spin column (QIAuick PCR Purification Kit, Qiagen). The qRT-PCR reactions were performed in a volume of 10 μl with 5 μl t (Applied Biosystems, Texas, USA), 0.4 μl forward and reverse primer (10 μM), 3.2 μl ddH_2_O, and 1 μl of cDNA (approximately 2 ng), under the following conditions: 95 °C for 3 min, followed by 40 cycles of 95 °C for 10 s and 60 °C for 10 s. The relative expression level and fold change (FC) of each candidate gene relative to the susceptible strains was calculated using the ΔΔcT method [33] after normalisation with two housekeeping genes, RPS3 (ribosomal protein S3) and the 60S ribosomal protein L8. All primer sequences and their origins are shown in Additional file 2: Table S1.

### Statistical analyses

Effects of strains, age, and exposure duration or of strain and synergist exposure were analysed using generalised linear models with binomial link functions in SPSS v22. Pearson chi-square tests were used to assess associations between *kdr* mutations and phenotypes in genotypic and allelic tests, with odds ratios used to measure effect size. Error bars represent 95% confidence intervals calculated using the method of Wilson, with continuity correction [34]. Haploview [35] was used to estimate haplotype frequencies and perform haplotype association tests for the *kdr* mutations. Two-tailed t-tests were used to compare candidate gene expression levels between strains: significance was accepted only when detected between a resistant strain and both susceptible strains.

## Results

### Phenotypic resistance

**Fig. 2:**
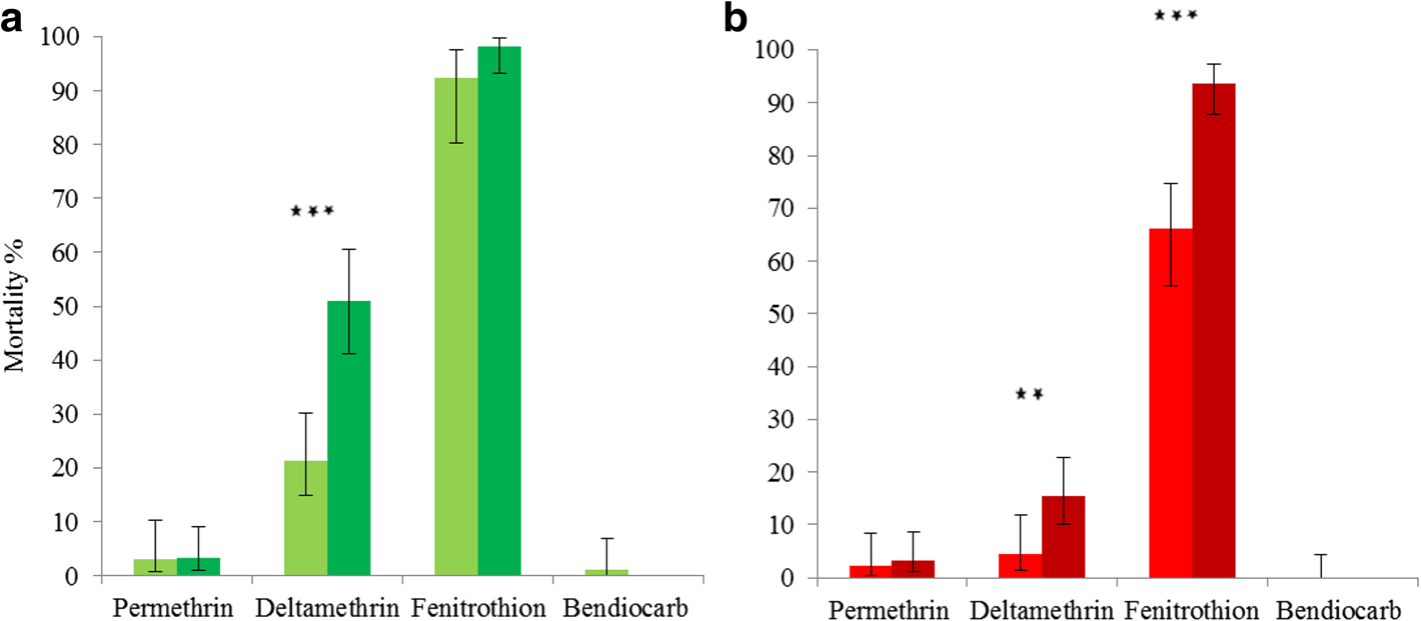
Susceptibility status of female *Ae. aegypti* to insecticides in 60 min bioassays with exposure to permethrin, deltamethrin, fenitrothion and bendiocarb. **a** Jeddah laboratory strain (*light green*) and field strain (*dark green*). **b** Makkah laboratory strain (*light red*) and field strain (*dark red*). Statistical significance is indicated by *P < 0.05, **P < 0.01 and ***P < 0.001. Error bars are 95% confidence intervals

**Fig. 3:**
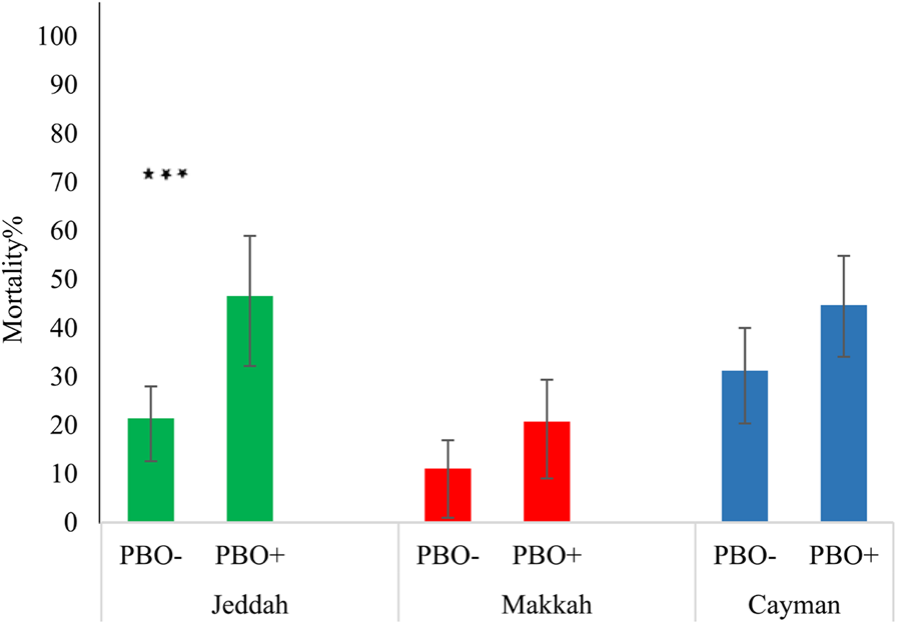
Deltamethrin 60 min bioassays with and without 60 min pre-exposure to the synergist PBO (PBO+, PBO- respectively). Statistical significance is indicated by *P < 0.05, **P < 0.01 and ***P < 0.001. Error bars are 95% confidence intervals

Bioassays on field and laboratory strains of female *Ae. aegypti* from Jeddah and Makkah indicated a high prevalence of resistance to permethrin and deltamethrin, and also to bendiocarb (tested in F1 females only). Qualitatively, results from the lab strains and field samples were comparable, despite their differing also in collection date and precise sample location. In some cases, mortalities were significantly higher in the field than F1 females (Fig. 2). A high prevalence of resistance to each pyrethroid was found in both Jeddah and Makkah and was especially pronounced for permethrin assays in which fewer than 10% of exposed females died. Similarly, mortality from bendiocarb exposure (tested in F1 females only) was negligible in each strain. For fenitrothion, assays on field samples revealed suspected evidence of resistance (mortality 90–97%) in Makkah, or confirmed resistance in the Makkah F1 strain, whereas in Jeddah field and F1, the results were more consistent and suggested borderline resistance (F1 = 92%; field = 98%). Although very similar for permethrin and bendiocarb, and equivocal for fenitrothion (owing to F1 *vs* field variation in Makkah), the prevalence of deltamethrin resistance was higher in Makkah than Jeddah females, whether tested on either F1 (*χ* ^2^ = 12.26, *df* = 1, *P* = 0.0005) or field females (*χ* ^2^ = 35.94, *df* = 1, *P* < 0.0001). PBO bioassays significantly improved susceptibility to deltamethrin (Fig. 3; Additional file 2: Table S2) and whilst the GLiM strain × PBO interaction term was not significant, only in the Jeddah strain (*χ* ^2^ = 12.17, *df* = 1, *P* = 0.0005), was the mortality significantly elevated (Makkah F1 strain, *χ* ^2^ = 2.26, *df* = 1, *P* = 0.13; Cayman strain; *χ* ^2^ = 3.41, *df* = 1, *P* = 0.065).

**Fig. 4:**
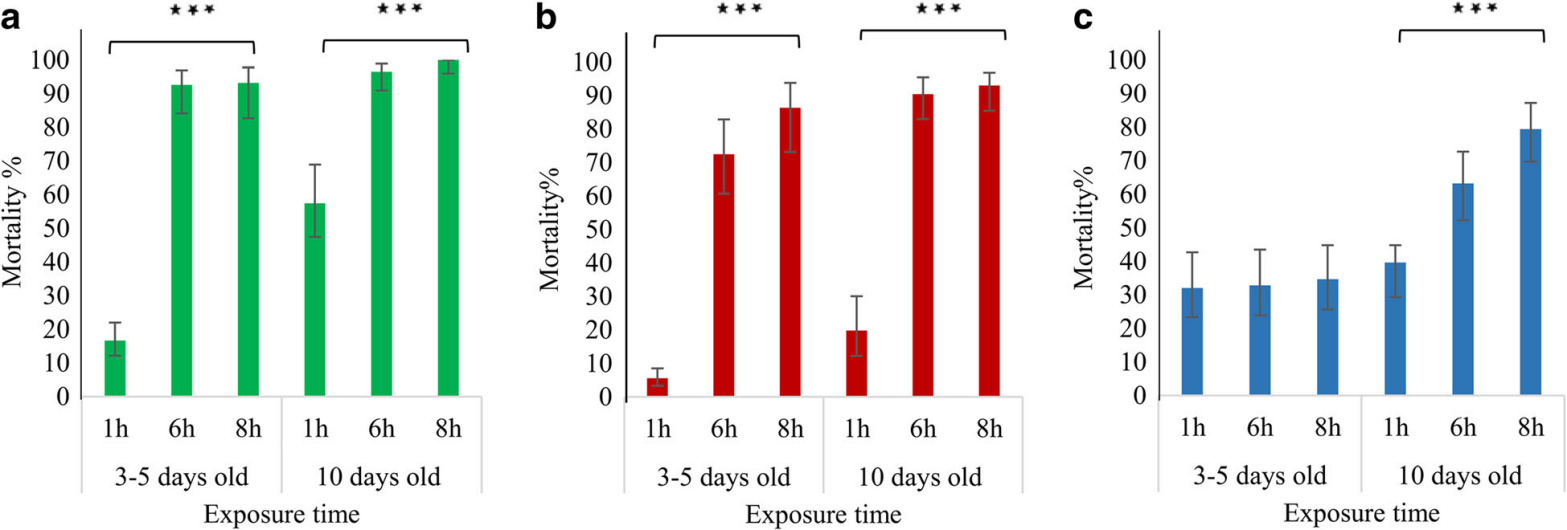
Impacts of age and exposure duration on deltamethrin survivorship in **a** Jeddah (*green*), **b** Makkah (*red*) and **c** Cayman (*blue*). Statistically significant variation among exposure times (ANOVA) is indicated by *P < 0.05, **P < 0.01 and ***P < 0.001. Error bars represent the 95% confidence intervals

The level of mortality and its dependence on female age was assessed in each of the three strains by exposing either 3–5 day-old or 10 days-old to deltamethrin for progressively longer periods of (1 h, 6 h, 8 h). Strain, age, and exposure were all significant in the GLiM, but effects on mortality were not straightforward, as evident from the significance of all three two-way interaction terms (Fig. 4; Additional file 2: Table S3). In both Saudi strains, the mortality was low following short exposure, though somewhat higher in older females, but mortality was much higher after longer exposures, irrespective of age (70–100%). In contrast, young females of the reference resistant strain Cayman exhibited no difference in mortality across exposure durations but when older females were tested longer exposures induced greater mortality (Fig. 4).

### Molecular mechanisms

DNA sequencing from wild (F0) mosquitoes of Jeddah and Makkah populations revealed three *kdr* substitutions in the *Vgsc*: S989P, V1016G and F1534C but neither of the I1011M or I1011V mutations (GenBank Accession Nos. S989P_V1016G: KY626180–KY626197, GenBank Accession Nos F1534C: KY046222–KY046237). The V1016G and S989P mutations were in perfect linkage disequilibrium (LD) in the Jeddah (*n =* 26) and Makkah (*n* = 15) F0 collections, with allele frequency estimates of 0.46 (95% CI: 0.33–0.59) and 0.67 (95% CI: 0.49–0.81), respectively. The allele frequency of 1534C was 0.5 (95% CI: 0.37– 0.63) in Jeddah and 0.4 (95% CI: 0.25–0.58) in Makkah and F1534C was in strong, but imperfect, repulsive LD with S989P/V1016G (*r* ^2^ = 0.59 in Jeddah and 0.87 in Makkah), with five Jeddah females and one Makkah female possessing one chromosome each with the triple mutant (989P + 1016G + 1534C) haplotype (Additional file 3: Table S4).

To evaluate impacts of the mutations on deltamethrin survival, haplotypes (estimated from genotypic data) of mosquitoes killed by duration WHO susceptibility bioassays (< 20% mortality in each strain) were compared with survivors of long 4-6 h bioassays (≥ 60% mortality in each strain). Four haplotypes were estimated in the two populations (Table 1) of which: 989P + 1016G + F1534 was strongly associated with resistance and S989 + V1016 + 1534C with susceptibility. The triple mutant 989P + 1016G + 1534C was somewhat more common in susceptible females, but the estimated frequencies were very low. Six distinct and one ambiguous triple-locus genotype could be discerned though not all were present in both Makkah and Jeddah. In concordance with the haplotypic tests, the double mutant genotype 989P/P + 1016G/G + 1534 F/F and single mutant genotype 989S/S + 1016 V/V + 1534C/C (numbers 1 and 2 in Table 2) differed strongly in the effect on resistance (*χ* ^2^ = 27.7, *df* = 1, *P* < 0.0001; OR = 79.2, 95% CI: 12–522). Though relatively rare, none of the six individual possessing genotypes which must have contained a triple mutant allele (i.e. 989P + 1016G + 1534C; genotype numbers 6 and 7) survived exposure, suggesting a lack of resistance conferred by this allele when heterozygous. Interestingly the triple heterozygote genotype (number 5) showed significant association with resistance (*χ* ^2^ = 17.4, *df* = 1, *P* < 0.0001; OR = 29.2, 95% CI: 5–164), which was approximately 2.7 times lower than the most resistant genotype (number 1), though not significantly so (*P* = 0.36).

**Table 1:** Estimated haplotype frequencies and their association with deltamethrin resistance in *Ae. aegypti* from each strain

	Jeddah			Makkah		
Haplotype	Susceptible	Resistant	*P*-value	Susceptible	Resistant	*P*-value
989P/1016G/F1534	0.187	0.628	6 × 10^-6^	0.143	0.853	2 × 10^-8^
S989/V1016/1534C	0.655	0.338	0.002	0.786	0.147	4 × 10^-7^
S989/V1016/F1534	0.079	0.030	0.319	0.036	0.000	0.269
989P/1016G/1534C	0.079	0.004	0.096	0.036	0.000	0.269

**Table 2:** Triple-locus *kdr* genotypes, shown as amino acids at codons 989, 1,016 and 1,534 and their frequencies in mosquitoes surviving a long (4–6 h) deltamethrin exposure or killed by a 1 h exposure

		Jeddah		Makkah		Combined				
Genotype		Alive	Dead	Alive	Dead	Alive	Dead	Mortality	LCL	UCL
1	PGF/PGF	7	3	12	0	19	3	0.14	0.04	0.36
2	SVC/SVC	2	16	0	9	2	25	0.93	0.74	0.99
3	SVF/SVC	0	4	0	0	0	4	1.00	0.40	1.00
4	SVF/PGF	1	1	0	1	1	2	0.67	0.13	0.98
5	SVF, SVC/PGF, PGC	9	3	5	3	14	6	0.30	0.13	0.54
6	SVC/PGC	0	3	0	1	0	4	1.00	0.40	1.00
7	PGF/PGC	0	2	0	0	0	2	1.00	0.20	1.00

Mutant amino acids are shown in bold type. LCL and UCL are 95% binomial confidence limits. Note that for genotype 5 alleles could not be determined unambiguously and alternates are shown

**Fig. 5:**
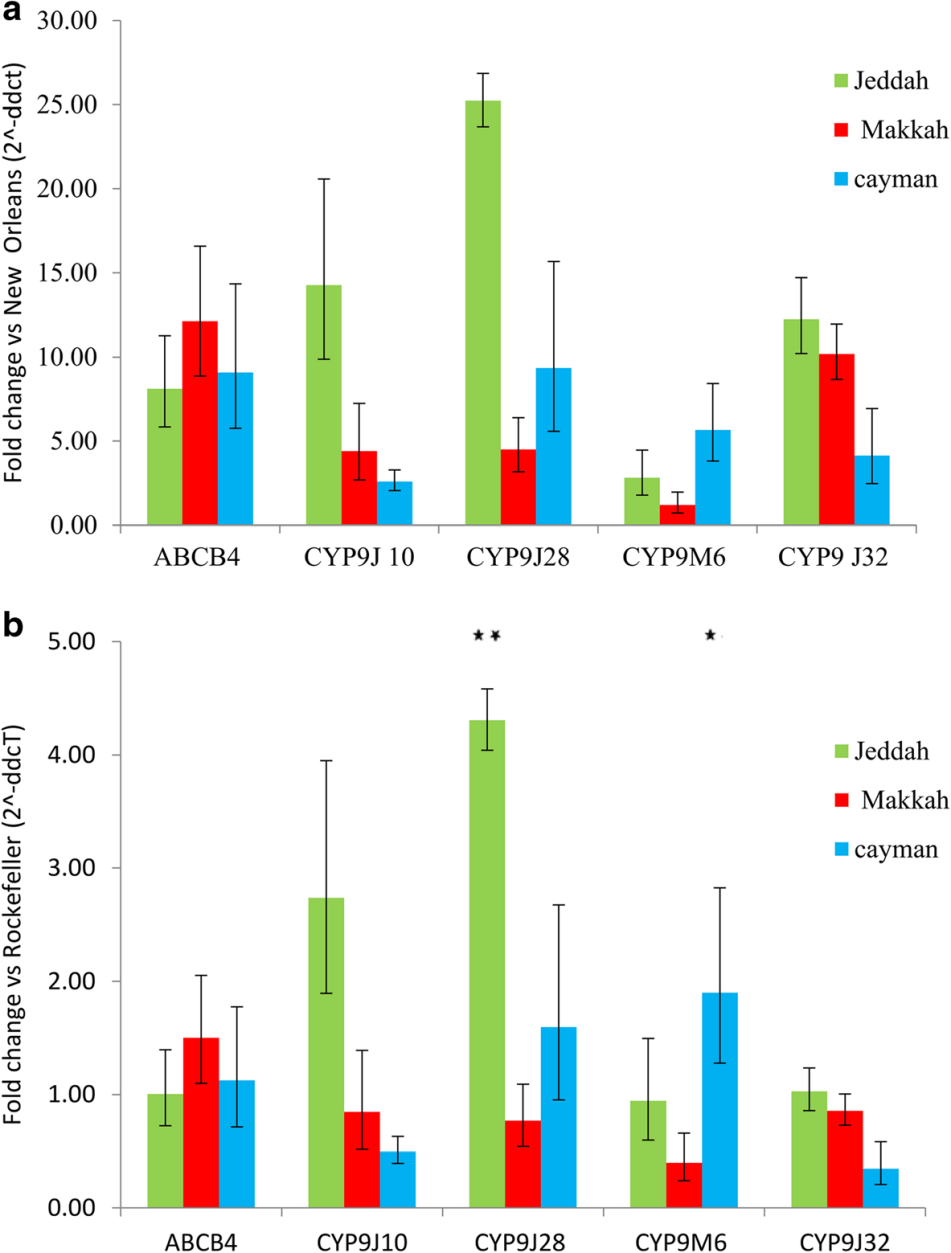
Quantitative PCR analysis of candidate genes. Relative-fold changes compared to two susceptible strains **a** New Orleans, **b** Rockefeller are shown following normalisation to two endogenous reference genes. Error bars represent 95% confidence intervals. Significance is indicated for Rockefeller only where New Orleans is also significant (*P < 0.05, **P < 0.01)

Quantitative real-time PCR in all strains (each unexposed to insecticide) showed several genes to be more highly expressed than in the New Orleans colony. However, when considering comparisons with Rockefeller, fewer of the candidate genes were significantly upregulated (Fig. 5). In the Makkah strain only *ABCB4* neared significance, and in the Jeddah strain, *CYP9J28* and *CYP9J10* were significantly and consistently overexpressed to high levels, and in the Cayman strain, the *CYP9M6* gene was over-expressed compared to both susceptible colonies. In each case, expression levels of the four P450 genes were lower in Makkah than in Jeddah.

## Discussion

Physiological resistance to pyrethroids in adult *Ae. aegypti* has now been detected around the globe with reports from Brazil, Mexico, Thailand, China, Grand Cayman, Latin America, Indonesia and Malaysia [26]. Reports of resistance from the Middle-Eastern region involve studies from the Ibb region of Yemen [36], from Jazan in Saudi Arabia [11] approximately 500 km north, and from Makkah [10], a further 700 km north. WHO bioassay results in each case were similar, with mortalities ranging from 75–93% to different pyrethroids. The previous study from Makkah [10] performed collections in 2008 and our data, which was gathered using the same methodology, suggests that in the intervening years, resistance to both permethrin and deltamethrin (<20% mortality in our results) may have increased substantially though we note that in the absence of repeated samples, consistency of the bioassay data, and thus stability of this longer-term variation, cannot be assessed. Interestingly, despite the proximity of the sites, − Jeddah is less than 100 km from Makkah - standard 60 min assay deltamethrin resistance was significantly lower in Jeddah, although was still higher than in the Cayman reference resistant strain. These observations demonstrate a worrying temporal increase in resistance in Makkah together with unpredictability in the spatial scale over which resistance may vary, suggesting the involvement of local pressures rather than wider features of *Ae. aegypti* population genetic structure. In common with previous reports from Jazan province [11], Makkah and Jeddah strains showed high resistance to carbamate (benidocarb), but mortality following exposure to the organophosphate fenitrothion was only slightly reduced.

Age may also be an important factor in resistance and is crucial because older mosquitoes are more likely to transmit disease. In common with previous studies on *Ae. aegypti* and other mosquitoes [37, 38] ten-day-old females from Makkah and Jeddah were significantly more susceptible to deltamethrin than 3–5 days olds, though with a standard 60 min exposure the majority still survived. With long exposure times of six or eight hours up to 30% survival was observed, demonstrating that not only are most of the population classed as resistant (from 60 min bioassay data), but a small proportion is highly resistant, even when tested as older females. Surprisingly, given the slightly lower prevalence of resistance at 60 min, Cayman females of either age class survived long exposures better, suggesting a dissociation between prevalence and level of resistance, as documented in *Anopheles gambiae* [39].

Females from Jeddah suffered a significant increase in deltamethrin mortality, which more than doubled following pre-exposure to PBO. For Makkah (and Cayman) the increase was slight and not significant in either case, although the difference among populations was not large enough for detection of statistically significant heterogeneity. This contrasts with the almost complete synergy of deltamethrin by PBO (from approximately 5 to 98% mortality) reported by Bingham et al*.* in the Nha Trang *Ae. aegypti* strain from Vietnam [40], which, together with overexpression of the strongly pyrethroid-metabolising gene *CYP9J32* [14] relative to the susceptible Bora Bora strain, was interpreted as evidence for a dominant role of CYP450 enzymes in deltamethrin resistance. While the impact of PBO was clear, some caution is required in causal links with CYP450s because, while the action of P450s can be blocked, as demonstrated directly in vivo in *Ae. aegypti* [16] other effects such as reduced cuticular penetration may occur [41]. Moreover, comparison with a single susceptible strain may be problematic, because of variation in expression levels unlinked to resistance. In our gene expression results, most candidate genes including *CYP9J32*, were overexpressed relative to New Orleans, but far fewer in comparison with the Rockefeller strain, although both are susceptible to pyrethroids. It is, however, interesting to note that in Jeddah, which showed the highest PBO synergism, two candidate CYP450s were overexpressed, of which both *CYP9J10* and *CYP9J28* are frequently upregulated in resistant strains, and the latter metabolises pyrethroids [14], suggesting some involvement in resistance. In Cayman, which showed more limited (and marginally non-significant) synergism, *CYP9M6*, also a known pyrethroid metabolizer [16] was consistently overexpressed, though at a moderate level, whereas in Makkah, in which the highest prevalence of deltamethrin resistance but no PBO synergy was observed, no candidate genes were significant. Taken together, these results suggest that, despite an understandable concentration on the link between CYP450s and pyrethroid resistance, upregulation of genes with proven metabolic capacity may not translate into higher resistance, emphasising the need to consider alternative mechanisms.

Three *kdr* substitutions were detected at high frequency in the *Vgsc* (S989P, V1016G, and F1534C) in wild females from Jeddah and Makkah. Each of these has been linked to pyrethroid resistance [42–44], but none have previously been identified in the Middle Eastern region. The 1011 M/V mutation described in Latin America, Mexico and French Guiana [21, 45, 46] was absent. The S989P mutation was in perfect linkage disequilibrium with V1016G as observed in previous research [9, 42, 47], though this appears to be the first reported occurrence of S989P polymorphism outside of Asia. In both Makkah and Jeddah the 1016G + 989P haplotype was very strongly associated with survival of long deltamethrin exposures, supporting results from in vitro expression of the *Vgsc* mutations in *Xenopus* oocytes [48] and field demonstration in Thailand [9] that this combination of mutations works additively to generate high-level resistance. We detected strong repulsive linkage disequilibrium between 989S + 1016 V/989P + 1016G and F1534C, which has also been observed in Thai populations of *Ae. aegypti* [9], and is likely to at least partially explain the lack of association of the F1534C mutation with deltamethrin resistance. The 1534C mutation alone confers permethrin resistance [48, 49] and while in combination with 989P and 1016G in vitro confers strong deltamethrin insensitivity, this has yet to be demonstrated in the field. Indeed none of the six individuals possessing the triple mutant haplotype (989P + 1016G + 1534C) survived deltamethrin exposure. However, as also demonstrated in *Ae. aegypti* from Thailand, the triple heterozygous genotype S/P989 + V/G1016 + F/C1534 significantly improved deltamethrin survival and was relatively common, suggesting an important intermediate step for otherwise recessive mutations [50]. Triple mutant haplotypes have now been detected at low frequency in in Myanmar [47], Java [44], and now in Saudi Arabia, and the potential for combined impact of the three *Vgsc* mutations must remain a cause for concern. TaqMan qPCR assays provide useful tools for high throughput screening for the presence of F1534C in addition to the other mutations. The Cayman strain we used has a high frequency of the 1534C mutation in addition to the V1016I substitution [23], both of which are common in Latin America [26], and when combined have been shown to increase the survival rate to deltamethrin in Brazilian and Mexican populations [46, 51]. While the 1534C mutation is widespread, our study contributes to knowledge of the boundaries for each of the V1016 mutations, with the most westerly report of the 1016G mutation to date. Interestingly, the 1016I mutation has recently been detected in Ghana [27], alongside the F1534C substitution, suggesting a possible contact zone between the 1016G and 1016I mutations located between Saudi Arabia and West Africa.

## Conclusion

In this study, a high level of resistance to pyrethroids was found in Jeddah and Makkah, with significantly higher resistance in the latter. Resistance in Makkah appears to have increased in comparison to the previous published survey from 2008, perhaps as a result of high usage of pyrethroids in local vector control programmes. The moderate or very limited impact of PBO, especially in the more resistant Makkah population which also lacked significant P450 overexpression, suggests that *kdr* mutations, perhaps in combination with other as yet unknown mechanisms, are more significant here than CYP450 based metabolic resistance. Possible differences in the contribution of contrasting resistance mechanisms between populations may arise from different histories of insecticide usage for vector control, in addition to informing future control options.

## Additional files

Additional file 1: Figure S1. Locations of *Ae. aegypti* larval collections of in Jeddah and Makkah in March-April 2016. (DOCX 213 kb)

Additional file 2: Table S1. List of primer sequences for qRT-PCR. **Table S2.** Generalized Linear Model for the effects of strain and the addition of PBO synergist before deltamethrin exposure on mortality of *Aedes aegypti* females. **Table S3.** Generalized Linear Model for the effects of strain, age and duration of deltamethrin exposure on mortality of *Aedes aegypti* females. (DOCX 24 kb)

Additional file 3: Table S4. Genotypes of females surviving long (4 or 6 h) exposure or killed by short (1 h exposure). (XLSX 14 kb)

## Abbreviations

60S: Ribosomal protein L8
CCEs: Carboxyl/cholinesterase
CYP450s: Cytochrome P450s
DDT: Dichlorodiphenyltrichloroethane
GLiM: generalised linear model
GSTs: Glutathione-s-transferases
*Kdr*: Knockdown resistance
LSTM: Liverpool school of tropical medicine
PBO: Piperonyl butoxide
qRT-PCR: Quantitative reverse transcription polymerase chain reaction
RPS3: Ribosomal protein S3
*Vgsc*: Voltage-gated sodium channel
WHO: World health organization (WHO) paper-based bioassay
