# Supplementary figures and images for: Combined target site (kdr) mutations play a primary role in highly pyrethroid resistant phenotypes of Aedes aegypti from Saudi Arabia
Source: Parasit Vectors. 2017 Mar 27;10:161. doi: 10.1186/s13071-017-2096-6 (PMC5368989; doi:10.1186/s13071-017-2096-6)

**Figure S1.** Locations of *Ae. aegypti* larval collections of in Jeddah and Makkah in March-April 2016.


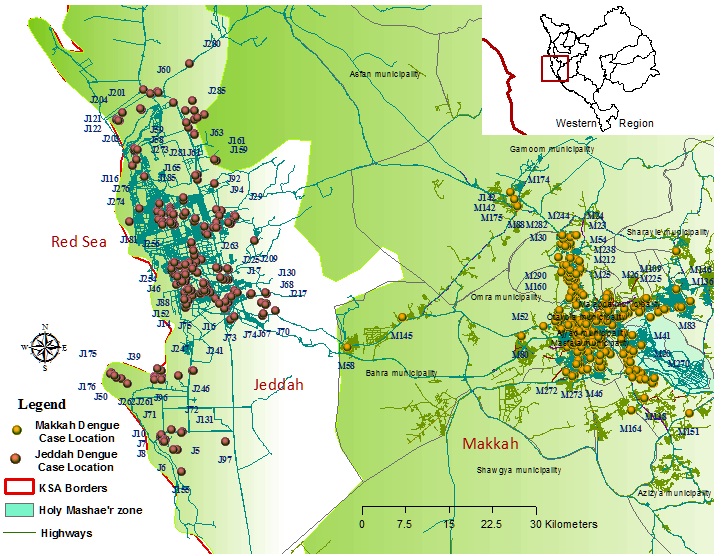

Supplement: Supplementary file 1 — Locations of Ae. aegypti larval collections of in Jeddah and Makkah in March-April 2016. (DOCX 213 kb) [file 13071_2017_2096_MOESM1_ESM.docx]
