# Supplementary material for: Combined target site (kdr) mutations play a primary role in highly pyrethroid resistant phenotypes of Aedes aegypti from Saudi Arabia
Source: Parasit Vectors. 2017 Mar 27;10:161. doi: 10.1186/s13071-017-2096-6 (PMC5368989; doi:10.1186/s13071-017-2096-6)
Supplement: Supplementary file 2 — List of primer sequences for qRT-PCR. Table S2. Generalized Linear Model for the effects of strain and the addition of PBO synergist before deltamethrin exposure on mortality of Aedes aegypti females. Table S3. Generalized Linear Model for the effects of strain, age and duration of deltamethrin exposure on mortality of Aedes aegypti females. (DOCX 24 kb) [file 13071_2017_2096_MOESM2_ESM.docx]

**Table S1.** List of primer sequences for qRTPCR

| Primer name | Primer sequence (5'–3') | Reference |
| --- | --- | --- |
| CYP9J10 F | ATCGGTGTTGGTGAAAGTTCTGT | [1] |
| CYP9J10 R | CATGTCGTTGCGCATTATCCC | [1] |
| CYP9J28 F | CCACTGACGTACGATGCGA | [1] |
| CYP9J28 R | GCCGATCAGTGGACGGAGC | [1] |
| CYP9J32 F | CGGTCCGCTTATGACGAAGAG | Grigolaki et al. unpublished |
| CYP9J32 R | TTTGTTCGCTCCGAAGAGTGG | Grigolaki et al. unpublished |
| CYP9M6 F | TCGGTGCACAATCCAAACAAC | [2] |
| CYP9M6 R | GTCGGGTACGACCAACGAAA | [2] |
| ABCB4 F | GAATGGCCGCATCTGCCAG | [1] |
| ABCB4 R | CGTTTCCTTGGGACCGAGCT | [1] |
| RPS3 F | AGCGTGCCAAGTCGATGAA | [2] |
| RPS3 R | GTGGCCGTGTCGACGTACT | [2] |
| Ae60sL8 F | CTGAAGGGAACCGTCAAGCAA | [3] |
| Ae60sL8 R | TCGGCGGCAATGAACAACT | [3] |

1. Bariami V, Jones CM, Poupardin R, Vontas J, Ranson H. Gene amplification, ABC transporters and cytochrome P450s: unraveling the molecular basis of pyrethroid resistance in the dengue vector, *Aedes aegypti*. PLoS Negl Trop Dis*.* 2012; 6(6).

2. Kasai S, Komagata O, Itokawa K, Shono T, Ng LC, Kobayashi M, et al. Mechanisms of pyrethroid resistance in the dengue mosquito vector, *Aedes aegypti*: target site insensitivity, penetration, and metabolism. PLoS Negl Trop Dis. 2014; 8(6):e2948

3. Grisales N, Poupardin R, Gomez S, Fonseca-Gonzalez I, Ranson H, Lenhart A, et al. Temephos Resistance in *Aedes aegypti* in Colombia Compromises Dengue Vector Control. PLoS Negl Trop Dis *.* 2013; 7(9):e2438.

**Table S2.** Generalized Linear Model for the effects of strain and the addition of PBO synergist before deltamethrin exposure on mortality of *Aedes aegypti* females

|  |  |  |  |
| --- | --- | --- | --- |
| Source | Wald χ2 | df | Probability |
| (Intercept) | 76.11 | 1 | 0.000 |
| strain | 18.87 | 2 | 0.000 |
| PBO | 13.78 | 1 | 0.000 |
| strain * PBO | 1.83 | 2 | 0.401 |
|  |  |  |  |

**Table S3.** Generalized Linear Model for the effects of strain, age and duration of deltamethrin exposure on mortality of *Aedes aegypti* females

|  |  |  |  |
| --- | --- | --- | --- |
| Source | Wald χ2 | df | Probability |
| (Intercept) | 90.28 | 1 | 0.000 |
| strain | 97.45 | 2 | 0.000 |
| age | 105.04 | 1 | 0.000 |
| exposure | 372.62 | 2 | 0.000 |
| strain * age | 9.16 | 2 | 0.010 |
| strain * exposure | 163.61 | 4 | 0.000 |
| age * exposure | 6.92 | 2 | 0.032 |
